# Unique inverse association between allergic rhinitis and periodontitis: a nationwide population-based study

**DOI:** 10.1038/s41598-022-23543-9

**Published:** 2023-05-08

**Authors:** Dae-Yeob Kim, Jae-Kwan Lee, Eun-Kyoung Pang, Seong-Ho Choi, Jong-Bin Lee

**Affiliations:** 1 Department of Periodontology, Ewha Womans University Mokdong Hospital, Seoul, Republic of Korea; 2 Department of Periodontology, Research Institute of Periodontal Regeneration, Yonsei University College of Dentistry, 50-1 Yonsei-ro, Seodaemun-gu, Seoul, 03722 Republic of Korea; 3 Department of Periodontology, College of Medicine, Ewha Womans University, Seoul, Republic of Korea; 4 Department of Periodontology and Research Institute of Oral Sciences, Gangneung-Wonju National University College of Dentistry, 7 Jukheon-gil, Gangneung, Gangwon-do 25457 Republic of Korea

**Keywords:** Dental diseases, Respiratory tract diseases

## Abstract

The increase in fine dust levels in the atmosphere has been associated with a growth in the incidence of environmental diseases, including allergic rhinitis (AR). Nasal obstruction caused by AR can impact the conditions in the oral cavity. The aim of this study was to determine the association between AR and periodontitis in the Republic of Korea. This study was based on data from the Seventh Korea National Health and Nutrition Examination Survey (KNHANES VII-1, 2016), which was conducted by the Korea Centers for Disease Control and Prevention. The study included 6129 adults older than 19 years. Sociodemographic information and medical variables including history of treatment of periodontitis (HTP) reflecting diagnosis of periodontitis and diagnosis of diseases such as AR were extracted from the data. HTP and AR were reported for 22.81 ± 0.84% (weighted percentage ± standard error) and 15.32 ± 0.63% of the studied population, respectively. A diagnosis of AR was reported for 11.07 ± 1.28% of those with HTP and for 17.55 ± 1.84% of those without HTP. From these, it was inferred that the prevalence of HTP was 1.536-fold higher in the non-AR group than in their counterparts with AR. Significant association was found between AR and HTP among those aged ≤ 64 years and the odds ratio (OR) of AR group for HTP was 0.62 (95% confidence interval:0.44–0.87; *P* = 0.0057). From this result, it can be inferred that patients diagnosed AR have lower risk of periodontitis.

## Introduction

Allergic rhinitis (AR), which is one of the more prevalent respiratory diseases, can cause sneezing, runny nose, postnasal drip, and nasal congestion concomitantly with eye symptoms such as redness and watery eyes^1^. Airborne allergens are generally considered to be the most common trigger^2^. Statistical data from the Korean Ministry of Health and Welfare indicate that the number of patients affected by AR in the Republic of Korea has increased over the past 10 years. This may in part be attributable to the recent finding from the Health Effects Institute in the USA that the annual average fine dust concentration in Korea ranked second to Turkey among nations in the Organization for Economic Co-operation and Development. Moreover, there has been a trend toward increasing fine dust concentration in Korea since 2011.

Nasal congestion, one of the common symptoms of AR, can induce the obstruction of nasal airway, leading to mouth breathing^1^, which has been reported to have adverse effects by changing composition of saliva and oral normal flora inducing gingival inflammation, halitosis, and an altered dentofacial growth pattern^3–5^. In particular, periodontitis, which is characterized by inflammation of the periodontal tissue (including gingiva and alveolar bone), is associated with a significantly higher gingival inflammation index in patients with mouth breathing than in their counterparts with closed-mouth breathing^5–7^. Thus, there appears to be a connection in AR patients between periodontal diseases and mouth breathing induced by nasal congestion. The oral cavity is anatomically adjacent to the nasal airway, which may allow conditions in one to impact those in the other. For example, especially for maxillary molars and sinus floor located in maxilla with anatomical proximity, odontogenic maxillary sinusitis is one of the common diseases^8^.

According to position paper from the ‘Journal of Periodontology’, the prevalence of periodontal disease in the adult population has been reported to be more than 20% including 50% of those who aged 55–64 suffering from moderate to severe periodontitis^9^. Periodontitis is a major cause of tooth loss among middle-aged people and older, and can influence quality of life while simultaneously conferring a large social cost for treatment^10^. In this context, given that AR is an emerging and tentative risk indicator for periodontitis, investigations of the association between AR and periodontitis may contribute to improved public welfare. There have been very few studies of this association, and there is no consistency among these studies. For example, Hung et al. reported that there was increased risk of periodontal disease in patients with AR in Taiwan^11^, whereas Friedrich et al. demonstrated an inverse association between periodontitis and respiratory allergies including AR^12^.

The purpose of the present study was to determine whether an association exists between AR and periodontitis among the population of Korea, adjusting for the impact of exogenous variables, which can have influence on the outcome variable, including sociodemographic features, systemic health status, and oral hygiene behaviors.

## Results

### Characteristics of the study population

Based on previous studies, there may be an association between sociodemographic variables and factors related to systemic diseases, and periodontitis^11,13^. The characteristics of the study population, which are listed in Table 1, were well-balanced with respect to sex, with 49.75% being male. Mean age of study population was 46.93 years. Education level was classified into three groups: lower than high school, high school, and higher than high school, accounting for 22.54%, 27.44%, and 50.03% of the study population, respectively. The rate of alcohol consumption (defined as at least once per month) was 59.39%, and 22.62% were smokers. The most common systemic diseases were hypertension (HTN, 28.13%), diabetes mellitus (DM, 10.79%), and osteoporosis (OP, 6.23%); rheumatoid arthritis (RA), angina pectoralis, chronic obstructive pulmonary disease (COPD), and myocardial infarction were found in 1.72%, 1.60%, 0.98%, and 0.87%, respectively. The rate of auxiliary oral hygiene device use (AOHD) was 51.96%. A history of treatment for periodontitis (HTP) was recorded for 22.81% of the study population, and AR group took 15.32% of the study population.

**Table 1:** Characteristics of the study population.

Variable	Weighted%	SE
Female	50.25	0.59
**Education level**		
< High school	22.54	0.96
High school	27.44	0.91
High school <	50.03	1.39
**Income**		
1/4 Quadrant	25.70	1.20
2/4 Quadrant	24.60	0.94
3/4 Quadrant	24.87	0.83
4/4 Quadrant	24.83	1.35
Alcohol consumption (≥ once/month)	59.39	0.88
Smoking	22.62	0.83
DM	10.79	0.50
HTN	28.13	0.81
COPD	0.98	0.18
RA	1.72	0.16
OP	6.23	0.34
Myocardial infarction	0.87	0.13
Angina pectoralis	1.60	0.16
AOHD	51.96	0.94
HTP	22.81	0.84
AR	15.32	0.63
Age, years*	46.93	0.37
BMI, kg/m^2^*	24.00	0.07

*AOHD* use of auxiliary oral hygiene devices, *AR* allergic rhinitis, *BMI* body mass index, *COPD* chronic obstructive pulmonary disease, *DM* diabetes mellitus, *HTN* hypertension, *HTP* history of treatment for periodontitis, *OP* osteoporosis, *RA* rheumatoid arthritis, *SE* standard error.

*Weighted mean with standard error.

### Distribution of variables according to HTP

Table 2 presents the distribution of variables according to the presence (HTP group) or absence of HTP (non-HTP group). Education level (< high school), DM, HTN, OP, and age (≥ 65 years) were statistically significantly higher in the HTP group. A diagnosis of AR was recorded for a statistically significantly greater proportion of patients in the non-HTP group than in the HTP group (17.55% vs 11.07%, *P* = 0.0002). The proportions of subjects with HTP among those with (AR group) and without AR (non-AR group) were calculated as 0.1572 and 0.2416, respectively (Table 3). Thus, the risk of periodontal disease was 1.536-fold higher in the non-AR group than in AR subjects.

**Table 2:** Distribution of variables according to history of treatment for periodontitis.

HTP	No		Yes		*P*
Variable	Weighted%	SE	Weighted%	SE	
Female	50.81	1.04	49.33	2.04	0.5489
**Education level**					< 0.0001**
< High school	19.33	1.13	27.29	1.82	
High school	24.43	1.16	32.90	2.28	
High school <	56.24	1.64	39.81	2.54	
**Income**					0.1934
1/4 Quadrant	22.48	1.46	22.82	2.03	
2/4 Quadrant	22.25	1.22	25.59	1.95	
3/4 Quadrant	25.30	1.14	25.14	1.59	
4/4 Quadrant	29.97	1.71	26.45	2.28	
Alcohol consumption (≥ once/month)	60.59	1.06	56.84	1.98	0.0766
Smoking	20.89	1.02	21.28	1.81	0.8508
DM	9.18	0.66	16.47	1.43	< 0.0001**
HTN	25.19	0.98	36.53	1.78	< 0.0001**
COPD	1.05	0.29	1.17	0.44	0.8164
RA	1.98	0.30	2.48	0.54	0.4082
OP	5.54	0.48	10.22	1.01	< 0.0001**
Myocardial infarction	0.75	0.17	1.27	0.36	0.1442
Angina pectoralis	1.55	0.25	2.34	0.61	0.1832
AOHD	57.66	1.21	57.09	2.18	0.8121
Age ≥ 65 years	13.63	0.81	21.36	1.50	< 0.0001**
BMI, kg/m^2^*	23.84	0.09	24.28	0.15	0.007**
AR	17.55	1.00	11.07	1.28	0.0002**

*AR* allergic rhinitis, *AOHD* use of auxiliary oral hygiene devices, *BMI* body mass index, *COPD* chronic obstructive pulmonary disease, *DM* diabetes mellitus, *HTN* hypertension, *HTP* history of treatment of periodontitis, *OP* osteoporosis, *SE* standard error, *RA* rheumatoid arthritis.

*Weighted mean with standard error.

**There was statistically significant difference between the group with HTP and the group without HTP. Education level (*P* < 0.0001), DM (*P* < 0.0001), HTN (*P* < 0.0001), OP (*P* < 0.0001), Age (*P* < 0.0001), BMI (*P* = 0.007) and AR (*P* = 0.0002).

**Table 3:** Proportion of patients with a history of treatment for periodontitis among patients with (AR group) and without allergic rhinitis (non-AR group).

	HTP in AR group	HTP in non-AR group
Approximate proportion	0.1572	0.2416*

*AR* allergic rhinitis, *HTP* history of treatment of periodontitis.

*The proportion of patients with HTP was approximately 1.536 times higher in the non-AR group than in patients with AR.

### Evaluation of associations

**Figure 1:**
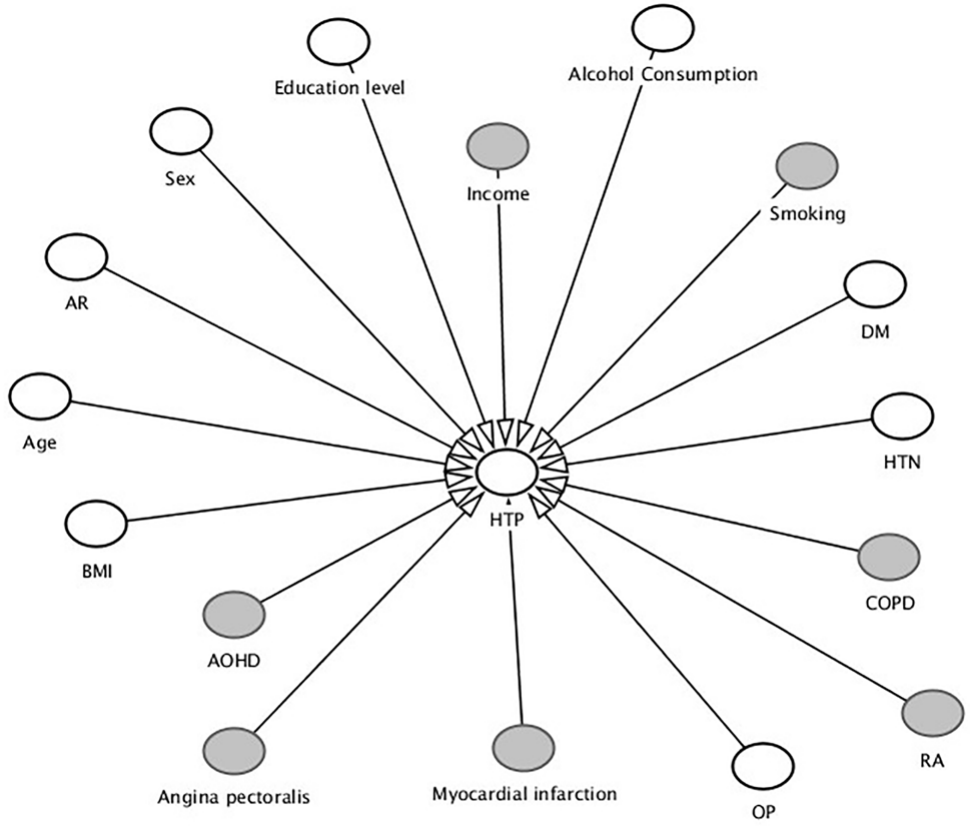
Variables included in this study. Line arrow suggests that each variable have association with periodontitis, supported by previous studies. Variables with white color are included in multiple logistic analysis, selected from univariate analysis. Variables with grey color are excluded from multiple logistic analysis. *AOHD* use of auxiliary oral hygiene devices, *AR* allergic rhinitis, *BMI* body mass index, *COPD* chronic obstructive pulmonary disease, *DM* diabetes mellitus, *HTN* hypertension, *HTP* history of treatment for periodontitis, *OP* osteoporosis, *RA* rheumatoid arthritis.

Associations between multiple factors and HTP were evaluated in two steps to consider the effect of potential exogenous variables of concern (Table 4). It has been suggested that one of the ways of control the exogenous variables is to put this variable into study design as an independent variable^14,15^. Univariate analysis was performed for each variable, revealing *P* values of < 0.1 for education level, alcohol consumption, DM, HTN, OP, age, body mass index (BMI), and AR (Fig. 1). Multiple logistic analysis using these variables and sex suggested that there were statistically significant associations between HTP and several of these variables, including education level (higher than high school), with an odds ratio (OR) of 0.70, and presence of OP or AR, with ORs of 1.48 and 0.68, respectively. Statistically significant association was not found from multiple logistic analysis between HTP and other variables such as alcohol consumption, DM, HTN, age and BMI.

**Table 4:** Results of univariate and multivariate analyses to adjust for exogenous variables.

Variable	Crude model				Multiple logistic analysis			
	OR	Lower 95% CL	Upper 95% CL	*P*	OR	Lower 95% CL	Upper 95% CL	*P*
Female*	0.94	0.78	1.15	0.5497	0.92	0.73	1.14	0.4272
**Education level**								
High school	0.95	0.74	1.23	0.7114	1.20	0.88	1.63	0.2502
High school <	0.50	0.40	0.63	< 0.0001**	0.70	0.51	0.96	0.027***
**Income**								
2/4 Quadrant	1.13	0.87	1.48	0.3616				
3/4 Quadrant	0.98	0.76	1.27	0.87				
4/4 Quadrant	0.87	0.68	1.11	0.2599				
Alcohol consumption (≥ once/month)	0.86	0.72	1.02	0.0791**	0.98	0.79	1.20	0.8121
Smoking	1.02	0.80	1.30	0.8504				
DM	1.95	1.51	2.52	< 0.0001**	1.33	1.00	1.78	0.051
HTN	1.71	1.43	2.04	< 0.0001**	1.26	1.00	1.60	0.0531
COPD	1.12	0.44	2.83	0.8168				
RA	1.26	0.73	2.18	0.4107				
OP	1.94	1.46	2.59	< 0.0001**	1.48	1.03	2.14	0.0355***
Myocardial infarction	1.71	0.82	3.55	0.1499				
Angina pectoralis	1.52	0.81	2.86	0.1878				
AOHD	0.98	0.81	1.18	0.8124				
Age ≥ 65 years	1.72	1.42	2.09	< 0.0001**	1.14	0.84	1.54	0.4029
BMI	1.04	1.01	1.06	0.006**	1.02	0.99	1.05	0.2061
AR	0.59	0.44	0.79	0.0004**	0.68	0.50	0.92	0.0126***

*AR* allergic rhinitis, *AOHD* use of auxiliary oral hygiene devices, *BMI* body mass index, *CL* confidence level, *COPD* chronic obstructive pulmonary disease, *DM* diabetes mellitus, *HTN* hypertension, *HTP* history of treatment of periodontitis, *OP* osteoporosis, *OR* odds ratio, *RA* rheumatoid arthritis.

*Sex was included in multiple logistic analysis.

**Variables with *P* < 0.1 in univariate analysis were included in multiple logistic analysis. In case of education level, both groups were included in multiple logistic analysis for accurate analysis although only higher than high school group had *P*-value under 0.1.

***The ORs for education level (Highschool <), OP, and AR versus HTP were statistically significant in multiple logistic analysis. High school < (*P* = 0.027), OP (*P* = 0.0355) and AR (*P* = 0.0126).

### Subanalysis based on the characteristics of the study population

One of the characteristics of this study population was the distribution of education level according to age (Table 5). More than 50% of the subjects aged at least 65 years did not graduate from high school. This was adjusted for by performing multivariate subanalysis with discrimination of subjects based on an age of 65 years (Table 6). A statistically significant association was found between AR and HTP among those younger than 65 years (OR = 0.62, *P* = 0.0057). In addition, the ORs for this association for education level (higher than high school) and OP among those aged younger than 65 years were 0.65 (*P* = 0.0241) and 1.99 (0.0108) with statistical significance, respectively. No such association was found for the older age group.

**Table 5:** Distribution of education level by age group.

Age group, years	< High school graduation			High school graduation			High school <		
	Frequency	Weighted%	SE	Frequency	Weighted%	SE	Frequency	Weighted%	SE
< 45	49	4.3286	0.6727	510	36.9988	1.7357	1674	70.2665	1.661
45–64	619	44.8652	1.5878	774	53.4869	1.7364	695	25.7942	1.4466
≥ 65	1071	50.8062*	1.5514	244	9.5143*	0.7271	166	3.9392*	0.4588

*SE* standard error.

*More than 50% of the population older than 65 years had a lower education level (< high school graduation).

**Table 6:** Results of multivariate subanalysis based on age.

Age < 65 years					Age ≥ 65 years				
Multiple logistic analysis					Multiple logistic analysis				
Variable	OR	Lower 95%CL	Upper 95%CL	*P*	Variable	OR	Lower 95%CL	Upper 95%CL	*P*
Female	0.92	0.71	1.18	0.5051	Female	1.01	0.68	1.50	0.9743
**Education level**					**Education level**				
High school	1.15	0.78	1.68	0.4812	High school	1.08	0.67	1.74	0.7553
High school <	0.65	0.44	0.94	0.0241*	High school <	1.22	0.72	2.09	0.4597
Alcohol consumption (≥ once/month)	0.92	0.73	1.16	0.4584	Alcohol consumption (≥ once/month)	1.28	0.88	1.87	0.198
DM	1.42	0.94	2.14	0.099	DM	1.17	0.76	1.80	0.4661
HTN	1.31	0.98	1.75	0.0658	HTN	1.03	0.72	1.49	0.8573
OP	1.99	1.18	3.38	0.0108*	OP	1.20	0.74	1.95	0.4592
BMI	1.01	0.98	1.05	0.4942	BMI	1.05	0.99	1.11	0.1069
AR	0.62	0.44	0.87	0.0057*	AR	1.31	0.69	2.49	0.4098

*AR* allergic rhinitis, *BMI* body mass index, *CL* confidence level, *DM* diabetes mellitus, *HTN* hypertension, *OP* osteoporosis, *OR* odds ratio.

*The OR for Education level (High school <), OP and AR over HTP was statistically significant in multiple logistic analysis among population younger than 65 years. High school < (*P* = 0.0241), OP (*P* = 0.0108) and AR (*P* = 0.0057).

## Discussion

The findings of these analyses of data from 6129 subjects extracted from the Seventh Korea National Health and Nutrition Examination Survey (KNHANES VII-1, 2016) demonstrated that for patients aged younger than 65 years, HTP was less prevalent among those with a diagnosis of AR, indicating that patients with AR had a lower risk of periodontitis. This finding is supported by that of a similar study by Friedrich et al., which suggested that there was an inverse association between periodontitis and allergic respiratory diseases^12^. Moreover, Grossi et al. reported a negative association between history of allergies and the severity of bone resorption based on a cross-sectional study of 1361 patients^16^.

The present study also revealed associations between HTP, education level, and presence of OP. This is in line with the finding in another study finding a significant inverse association between education level and risk of periodontitis^17^. Furthermore, OP is considered as a risk factor for the progression of preexisting periodontitis^18^.

These statistical phenomena can be interpreted in the context of the T-cell-mediated immune response. Upon recognizing an antigen, naïve T cells differentiate into several kinds of T cells, including T-helper (Th) cells and regulatory T cells (Treg)^19^. There are three types of Th cell: Th1, Th17, Th2, and Treg^19^.

The Th1/Th2 hypothesis is one of theories regarding the mechanism of immune regulation and is based on homeostasis between the activities of Th1 and Th2 cells^20^, in which they act as cross-inhibitors for each other, thus maintaining a balance in their activities^19,21^. Th1/Th2 immune responses can account for various diseases^22^. Th1-related cytokines are connected to autoimmune-related pathology^20^. They promote inflammation pathways through secretion of the cytokine interferon gamma (IFN-γ), which activates macrophages, in turn suppressing Th2 activity^19,20^. In some studies it was suggested that Th1 cells and IFN-γ contribute to the breakdown of periodontal tissue by stimulating monocytes and macrophages^23^. Meanwhile, Th2-related cytokines are involved in the genesis of allergic diseases^22^. Th2 cells inhibit Th1 cells via the production of interleukin (IL)-10; they also stimulate antibody formation by B cells through the production of IL-4 and IL-5^20,21^. One of the main roles of Th2 cells is the production of the immunoglobulin E (IgE)-synthesizing cytokines IL-4 and IL-13; IgE is involved in the allergic reaction^24^.

Periodontitis is regarded as an infectious pathology of periodontal tissue with several specific characteristics^25^. Subgingival pathogens can interact with and invade periodontal tissues^25^. Although bacterial pathogens are considered to initiate the periodontal disease, the host response appears to be related to destruction of gingival tissue and bone^26^. Invasion of these antigens can cause an inflammatory reaction and generation of immune responses, including innate and adaptive immune responses^26^. Periodontal tissue breakdown occurs mainly via cellular immune responses together with proinflammatory mediators such as tumor necrosis factor, IL-1β, and IL-17, which promote degradation of gingival tissue and bone resorption^19,27^. As a part of this mechanism, activated lymphocytes including Th1 and Th17 play important roles in the loss of bone through a RANKL-dependent mechanism^28^.

AR, a symptomatic pathologic state of the nose caused by exposure to allergens, is an IgE-mediated hypersensitivity reaction^29^. The pathogenesis of AR starts with the dendritic-cell-induced activation of Th2 cells, which themselves induce the production of IL-4 and IL-13, and ultimately IgE^30^. Specific IgE antibodies formed by B cells become attached to mast cells to enable cross-linking between the two^31^. This results in release of histamine, leukotrienes, and prostaglandins from mast cells, causing typical immediate reactions of AR such as sneezing, itching, and running of the nose or blockage of the nasal epithelium^30^. As mentioned above, overactivation of Th1 or Th2 cells can cause disease, and either pattern may inhibit the other^20^. In this context, AR with a Th2-dominant state may cause the down-regulation of the Th1 pathway, resulting in suppression of periodontal tissue destruction by proinflammatory cytokines.

Children account for almost 40% of all AR patients, with adults accounting for only 10–30%^32–34^. Conversely, several studies suggest that the prevalence of periodontitis increases with age^35–37^. The differences in the distribution of AR and periodontitis prevalence with age may support the explanation for the present findings. It can be hypothesized that diagnosed AR in young age may affect the occurrence of periodontitis in older age. In the context of immunology, a statistical association between periodontitis and Th2-related diseases such as asthma and atopic dermatitis is required to support that found in the present study between AR and periodontitis.

The Th1/Th2 theory is just one of the ways of explaining and understanding the process of immune regulation; however, that theory is still considered controversial, with limitations and discrepancies. Further research based on large-scale human studies are needed to support its validity^20^.

In conclusion, statistical analysis of data extracted from KNHANES VII-1 (2016) revealed that there was a significant association between AR and HTP, suggesting reduced risk of periodontitis in the AR group compared with the non-AR group particularly among those younger than 65 years. Higher education level was associated with decreased risk of periodontitis and presence of OP was associated with increased risk of periodontitis. The limitation of this study was that supplementary statistical analysis about secondary effects of variables should be included in multivariate statistical analysis although subanalysis based on the distribution of education level according to age was performed. In addition, the association between variables should be considered and reflected on statistical modeling. Variables in this study were selected from the KNHANES VII-1 (2016) raw data, which were known to have association with periodontitis according to the previous studies^37–39^. In Table 4, the crude model shows the result of univariate analysis. In this part, the association between each selected variable and HTP excluding other variables was checked. Based on this result, only variables with relatively higher statistical association with HTP were included in multiple logistic analysis. In this procedure, the association between HTP and each variable was evaluated, considering influences of exogeneous variables by including them as independent variables in multivariate analysis. Additionally, this study, as a cross-sectional study, was concentrated on association between two diseases based on ever prevalence of each disease in the specific time point. As mentioned above, the average diagnosis age of AR is younger than that of periodontitis in general, however, it cannot be demonstrated that all cases in this study have same sequence of onset. Therefore, data including the sequence of diagnostic experiences of both diseases in each individual are needed. There could be limitation on interpretation of cause-and-effect relation comparing to longitudinal study. To redeem this limitation and clarify statistical causation between two diseases, study including diagnosis age is necessary. Also, further longitudinal study is required to reveal the association between these factors and periodontitis. In this study, the association between AR and HTP in Korean population was suggested, for which there have been no research results so far. The limitation of this study will be supplemented through further studies.

## Methods

### Study population

This study was based on data from the KNHANES VII-1 (2016), conducted by the Korea Centers for Disease Control and Prevention^40^. The study included 6129 subjects**,** all of whom were adults older than 19 years. This study was approved by Institutional Review Board (IRB) of Ewha Womans University (approval No. EUMC 2020-02-033). The research was performed in accordance with relevant guidelines and regulations.

### Variables

Data on HTP, education level, income, alcohol consumption, smoking, AOHD, diagnosis of diseases such as AR, DM, HTN, COPD, RA, OP, and cardiovascular disease, and BMI were extracted from the data for the included individuals. AR was defined based on the self-report of diagnosis experience of AR by doctor ever. In case of HTP, it was also defined based on self-report of history of periodontal treatment. Investigation of medical history included in the present study were examined by questionnaire. Other factors except BMI such as age, sex, education level, and income were also examined by questionnaire. BMI was calculated by measuring height and weight of individuals. These variables except HTP and AR were selected as exogenous variables based on the previous studies suggesting that they had association with periodontitis^37–39^. As previous studies suggested, diagnosis of periodontitis was defined based on the HTP^41,42^. HTP included periodontal treatment other than scaling such as subgingival curettage, periodontal flap operation, and gingivectomy. Education level was divided into three groups: lower than high school, high school, and higher than high school. Income was classified into four grades (quartiles). Alcohol consumption was defined as a history of alcohol intake on one or more occasion per month within a 1-year period. AOHD, including floss, interdental brush, mouthwash, electric toothbrush, water flosser, tongue cleaner, and end-tuft brush, was ascertained by questionnaire (the “not using” group included only subjects who did not use any AOHD).

### Statistical analysis

Statistical estimations were made for the Korean population based on samples from KNHANES VII-1 using a complex sample design. Statistical analysis of this study was proceeded according to the analysis guideline from Korea Centers for Disease Control and Prevention. The sample weights, assigned to each variable, were set for sample participants to reflect the Korean population. Weighted percentages were used to express the proportions of each variable among the total population. Proportion of patients with HTP among patients with and without AR was calculated based on weighted percentage of HTP and AR group. Multiple logistic regression analysis was used to analyze associations between HTP and the other variables, with adjustment for exogenous variables, providing ORs. Univariate analysis was performed before multiple logistic regression and only variables with P value less than 0.1 were included in multiple logistic regression. Confidence level for multiple logistic regression analysis was set to 95%. P-value less than 0.05 was chosen as threshold for statistical significance. Additional sub-analysis, dividing population into two group based on age 65, was performed because population with lower education level was concentrated in aged over 65 group. There was no remarkable uneven distribution among other variables. SAS for Windows (version 9.4, SAS Institute, Cary, NC, USA) was utilized.

## Data Availability

The raw data used in this study was from the KNHANES VII-1 (2016), conducted by the Korea Centers for Disease Control and Prevention, available on website (https://knhanes.kdca.go.kr/knhanes/sub03/sub03_02_05.do). The data that support the findings of this study are available from the corresponding author upon reasonable request.
